# Lipid Metabolism and Ferroptosis

**DOI:** 10.3390/biology10030184

**Published:** 2021-03-02

**Authors:** Ji-Yoon Lee, Won Kon Kim, Kwang-Hee Bae, Sang Chul Lee, Eun-Woo Lee

**Affiliations:** 1 Metabolic Regulation Research Center, Korea Research Institute of Bioscience and Biotechnology (KRIBB), Daejeon 34141, Korea; sy92rhea@kribb.re.kr (J.-Y.L.); wkkim@kribb.re.kr (W.K.K.); khbae@kribb.re.kr (K.-H.B.); 2 Department of Functional Genomics, University of Science and Technology (UST), Daejeon 34141, Korea

**Keywords:** ferroptosis, lipid peroxidation, polyunsaturated fatty acids, GPX4, lipoxygenase

## Abstract

**Simple Summary:**

Ferroptosis is a type of cell death, which is morphologically and mechanistically distinct from other type of cell death pathways such as apoptosis and necroptosis. Lipid peroxidation is a hallmark of ferroptosis and directly destroys cellular membranes, thereby causing ferroptosis. Since lipid peroxidation, which induces ferroptosis, occurs in polyunsaturated fatty acid on specific phospholipids, various lipid metabolic pathways are involved in lipid peroxidation and ferroptosis. Besides, various metabolic and signaling pathways directly and indirectly regulate lipid peroxidation and ferroptosis. Since ferroptosis is associated with a variety of human diseases such as cancer, myocardial infarction, atherosclerosis, kidney diseases, liver diseases, and neuronal diseases, a better understanding of the regulatory mechanisms of ferroptosis can provide insights and treatment strategies for related diseases.

**Abstract:**

Ferroptosis is a type of iron-dependent regulated necrosis induced by lipid peroxidation that occurs in cellular membranes. Among the various lipids, polyunsaturated fatty acids (PUFAs) associated with several phospholipids, such as phosphatidylethanolamine (PE) and phosphatidylcholine (PC), are responsible for ferroptosis-inducing lipid peroxidation. Since the de novo synthesis of PUFAs is strongly restricted in mammals, cells take up essential fatty acids from the blood and lymph to produce a variety of PUFAs via PUFA biosynthesis pathways. Free PUFAs can be incorporated into the cellular membrane by several enzymes, such as ACLS4 and LPCAT3, and undergo lipid peroxidation through enzymatic and non-enzymatic mechanisms. These pathways are tightly regulated by various metabolic and signaling pathways. In this review, we summarize our current knowledge of how various lipid metabolic pathways are associated with lipid peroxidation and ferroptosis. Our review will provide insight into treatment strategies for ferroptosis-related diseases.

## 1. Introduction

Reactive oxygen species (ROS), including superoxides, hydroxyl radicals, hydrogen peroxide and lipid peroxides, are byproducts of aerobic metabolism and are oxygen-carrying molecules with reactive properties [1]. ROS can be generated in cells by various enzymes, such as NADPH oxidases (NOXs), lipoxygenases (LOXs), enzymes of cytochrome P450 (CYP450s), and cyclooxygenases (COXs) [2]. Excessive amounts of ROS are toxic to cells, directly damaging cellular components and leading to cell death, but cells have a defense mechanism against oxidative stress that directly or indirectly eliminates ROS [3]. Failure of the antioxidant mechanism can lead to the development of various degenerative diseases, such as neurodegenerative diseases and myocardial infarction [4,5,6]. On the other hand, non-toxic ROS act as signaling molecules involved in cellular processes such as cell cycle progression, genetic instability, epithelial-mesenchymal transition (EMT), and angiogenesis. Therefore, it is important to understand the role of ROS in order to develop treatment strategies for ROS-related diseases.

**Figure 1:**
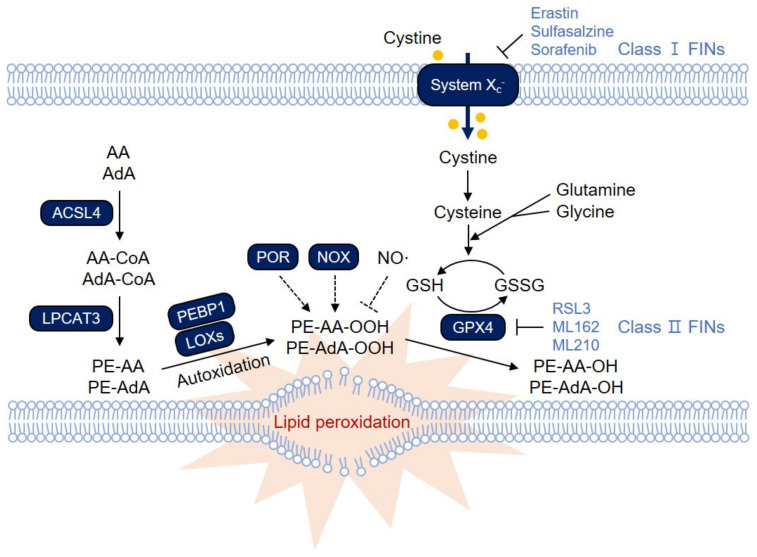
The ferroptosis signaling pathway. Polyunsaturated fatty acids (PUFAs) in membrane phospholipids undergo lipid peroxidation, which directly destroys the cellular membrane, thereby causing necrotic cell death via ferroptosis. Glutathione Peroxidase 4 (GPX4) reduces lipid peroxide to lipid alcohol by oxidizing glutathione (GSH), thereby protecting cells from ferroptosis under normal conditions. Inactivation of GPX4 or depletion of GSH therefore leads to massive lipid peroxidation and induces ferroptosis. Ferroptosis-inducing compounds (FINs) are categorized into two main groups: those that inhibit system x_c_^−^, thereby depleting GSH levels (class I FINs), and those that directly inhibit GPX4 (class II FINs). Among various membrane phospholipids, arachidonic acid (AA)- and adrenic acid (AdA)-containing phosphatidylethanolamine (PE) and phosphatidylcholine (PC) are the primary targets for lipid peroxidation. Acyl-CoA synthetase long-chain family member 4 (ACSL4) links free PUFAs to CoA, generating fatty acyl-CoA esters, which are eventually incorporated into PC/PE by lysophosphatidylcholine acyltransferase 3 (LPCAT3). PE-AA and PE-AdA can be oxidized by lipoxygenases (LOXs). LOX might require phosphatidylethanolamine-binding protein 1 (PEBP1) to induce lipid peroxidation on the membrane. In addition, other oxygenases, such as NADPH oxidases (NOXs) and cytochrome P450 oxidoreductase (POR), are known to contribute to lipid peroxidation. Lipid peroxidation is also mediated by nonenzymatic autoxidation, which is suggested to be the ultimate driver of ferroptotic cell death. In contrast, NO^•^ reacts with lipid peroxyradicals, thereby attenuating lipid peroxidation and ferroptosis.

Lipid peroxidation can directly damage cellular membranes, resulting in cellular dysfunction and cell death [7,8,9,10]. Therefore, lipid peroxidation has long been implicated in various diseases, such as atherosclerosis, neuronal diseases, and ischemic diseases [7,8,9,10]. Glutathione peroxidase 4 (GPX4) was originally identified as a phospholipid hydroperoxide glutathione peroxidase that reduces membrane-bound phospholipid hydroperoxide (Figure 1) [11,12]. Mice deficient in GPX4 exhibit embryonic lethality at day E7.5, suggesting an essential role of GPX4 in embryonic development [13]. Inducible GPX4 deletion results in massive lipid peroxidation and cell death in a LOX-12/15-dependent manner in vivo [14]. Neuron-specific deletion or inducible depletion of GPX4 causes neurodegeneration and acute renal failure, respectively, with an increase in lipid peroxidation, suggesting that GPX4 is a critical suppressor of lipid peroxidation and related pathologies [13,15].

Ferroptosis is an iron-dependent type of necrotic cell death characterized by the accumulation of lipid peroxides and was first introduced by Dixon et al. in 2012 [16]. Ferroptosis requires redox-active iron, which contributes to non-enzymatic lipid peroxidation (autoxidation) and enzymatic lipid peroxidation mediated by LOXs and thus can be inhibited by iron chelators such as deferoxamine (DFO) [16,17]. The accumulation of lipid peroxidation products in the cellular membrane can lead to membrane disruption and cell death mediated by ferroptosis. However, cells utilize endogenous lipophilic antioxidants and ferroptosis suppressor proteins such as GPX4 and apoptosis-inducing factor mitochondria-associated 2 (AIFM2), which has been renamed ferroptosis suppressor protein 1 (FSP1), to reduce lipid peroxides, thereby protecting cells from ferroptosis under normal conditions [18,19,20,21].

Ferroptosis can be induced by several ferroptosis-inducing compounds (FINs) [22,23]. Erastin is the first identified FIN that inhibits the cystine/glutamate antiporter (system x_c_^−^), thereby resulting in the depletion of glutathione (GSH), a cofactor for GPX4 (Figure 1) [16]. Inhibitors of system x_c_^−^ are classified as class I FINs and include sulfasalazine and sorafenib. Class II FINs such as RSL3, ML162, and ML210 covalently bind to and directly inhibit GPX4, thereby rapidly inducing ferroptosis (Figure 1) [24]. In addition, numerous compounds, such as FIN56 and withaferin A, have been identified as other classes of FINs [22,23]. In this review, we will focus on lipid metabolism, which governs cellular susceptibility to ferroptosis by regulating the intracellular composition of phospholipids and the process of lipid peroxidation.

## 2. Lipid Peroxidation and Ferroptosis

### 2.1. PE-AA and PE-AdA, the Most Susceptible Substrates for Lipid Peroxidation

Among the various ROS, lipid peroxides, including lipid peroxyradicals, are direct inducers of ferroptotic cell death [16]. Since the important role of lipid ROS in ferroptosis was revealed, there has been much interest in understanding which lipid species are preferred in the regulation of ferroptotic cell death. Comprehensive redox phospholipidomic liquid chromatography-tandem mass spectrometry (LC-MS/MS) analysis was applied to detect numerous oxygenated and non-oxygenated phospholipid species, including phosphatidylethanolamine (PE), phosphatidylcholine (PC), phosphatidylserine (PS), phosphatidylglycerol (PG), phosphatidylinositol (PI) and cardiolipin (CL), in cells [25]. Upon ferroptosis induction, most oxygenated phospholipid species are upregulated, suggesting that ferroptosis ultimately damages most membrane phospholipids [25]. By applying several criteria, Kagan et al. identified four molecular species of phospholipids, including doubly and triply oxygenated arachidonic acid (AA)- and adrenic acid (AdA)-containing PE species (C18:0/C20:4 and C18:0/C22:4), as the most crucial phospholipids for ferroptotic death signaling (Figure 1) [25]. Accordingly, these oxygenated phospholipid species also increased in Gpx4 KO mice but were reduced in mice treated with the ferroptosis inhibitor liproxstatin-1 [25].

In addition to PE, other phospholipids are also oxidized during ferroptosis [26,27]. In addition to PE, oxidized PS and PI are also elevated in ML162-induced ferroptosis in bone marrow-derived macrophages (BMDMs) [27]. Withaferin A also induces ferroptosis with a general increase in the oxygenated form of most classes of phospholipids. Notably, PG and PI are increased the most among other classes [27].

### 2.2. ACSL4 and LPCAT3 for Membrane Phospholipids

Since peroxidation on membrane phospholipids containing polyunsaturated fatty acids (PUFAs), but not free PUFAs, is responsible for ferroptotic cell death, enzymes involved in the incorporation of PUFAs into phospholipids play an indispensable role in ferroptosis. Acyl-CoA synthetase long-chain family member 4 (ACSL4) was first identified as an essential component for ferroptosis execution using a study employing a genome-wide CRISPR-based genetic screening system and microarray analysis of ferroptosis-resistant cell lines [25]. ACSL4 is a member of the ACSL family that links free long-chain fatty acids to CoA, generating fatty acyl-CoA esters, which are eventually transesterified into phospholipids (Figure 1) [28]. In particular, ACSL4 is known to preferentially recognize AA as a substrate [28]. Among the ACSL family enzymes, only ACSL4 impacts ferroptosis sensitivity in fibroblast cells [25]. ACSL4-deficient cells showed resistance to ferroptosis induced by GPX4 deletion or RSL3 treatment due to the reduced abundance of PE-AA and PE-AdA species [25]. Notably, ACSL4 is preferentially expressed in basal-like breast cancer cell lines, promoting ferroptosis, while it is often silenced in most luminal-type breast cancer cell lines that are resistant to ferroptosis, suggesting that ACSL4 determines ferroptosis sensitivity in breast cancer [25].

PUFA-CoA is then incorporated into phospholipids primarily in the endoplasmic reticulum (ER) by lysophosphatidylcholine acyltransferase 3 (LPCAT3), which preferentially uses PC and PE as substrates (Figure 1) [29]. LPCAT3 was identified through haploid gene screening in cells treated with nonapoptotic cell death-inducing small molecules [30]. In this screening, genes related to lipid metabolism, including ACSL4, were also identified, supporting the idea that PUFA metabolism plays a central role in ferroptosis. Knockdown of Lpcat3 causes resistance to RSL3-induced ferroptosis in mouse lung epithelial (MLE) cells and mouse embryonic cells, confirming the essential role of LPCAT3 in ferroptosis [25,30].

### 2.3. LOXs in Lipid Peroxidation

Lipid peroxidation occurs by two primary mechanisms: non-enzymatically spontaneous autoxidation and enzyme-mediated processes catalyzed by several enzymes [17,31,32,33,34]. In non-enzymatic autooxidations, free ferrous iron reacts with hydrogen peroxide (H_2_O_2_) and generates ferric iron and a hydroxyl radical [35,36]. The hydroxyl radical initiates the process of lipid peroxidation by abstracting hydrogen at the bis-allylic position of PUFAs [37]. LOXs are non-heme iron-containing dioxygenases that catalyze the stereospecific addition of oxygen onto PUFAs, such as AA and linoleic acids, resulting in lipid peroxidation (Figure 1) [38]. The U-shaped fatty acid binding channels of LOXs are hydrophobic, allowing PUFA substrates to easily access the LOXs. The oxygenation of AA occurs at a specific carbon position of AA depending on the depth of the U-shaped fatty acid binding channels [39,40]. The human genome contains six functional LOX genes (ALOXE3; Arachidonate lipoxygenase 3, ALOX5; Arachidonate 5-lipoxygenase, ALOX12; Arachidonate 12-lipoxygenase, 12S type, ALOX12B; Arachidonate 12-lipoxygenase, 12R type, ALOX15; Arachidonate 15-lipoxygenase, and ALOX15B; Arachidonate 15-lipoxygenase type B), each encoding a distinct LOX enzyme. However, the contribution of LOXs to lipid peroxidation and ferroptosis is still controversial. The 12/15-LOX-specific inhibitor PD146176, but not 5-LOX inhibitors (caffeic acid and MK886) or the COX inhibitor indomethacin, rescues *GPX4*^−/−^ cells from ferroptosis, suggesting that 12/15-LOX is responsible for ferroptosis [14]. In addition, *12/15-LOX*^−/−^ mouse embryonic fibroblasts (MEFs) are resistant to ferroptosis upon L-buthionine sulfoximine (BSO) treatment, which results in GSH depletion [14]. While the depletion of all LOXs can suppress erastin-induced ferroptosis, it fails to inhibit RSL3-induced ferroptosis in G401 renal carcinoma cells, so it is unclear whether LOXs also play a role in ferroptosis induced by GPX4 inhibition [17].

The p53 tumor suppressor has been reported to modulate ferroptosis. P53 can enhance ferroptosis by inhibiting the expression of SLC7A11, a component of system x_c_^−^, or promoting spermidine/spermine N1-acetyltransferase 1 (SAT1) and GLS2 expression [41,42,43,44]. On the other hand, p53 could suppress ferroptosis through the inhibition of dipeptidyl-peptidase 4 (DPP4) activity or the induction of Cyclin-dependent kinase inhibitor 1A/p21 (CDKN1A/p21) expression [20,45]. In particular, p53 upregulates 15-LOX, but not 5-LOX or 12-LOX, through SAT1, sensitizing cells to tert-butyl hydroperoxide (TBH)-induced ferroptosis, which is also attenuated by PD146176 [42]. RNAi-mediated loss-of-function screening showed that depletion of 12-LOX, but not that of the other five LOX isoforms, blocks TBH-induced ferroptosis in p53-overexpressing cells [46]. Interestingly, p53-mediated ferroptosis upon TBH is independent of ACSL4, and it remains unclear which phospholipids are responsible for ferroptosis [46].

Numerous studies have suggested that several LOX inhibitors, including zileuton (5-LOX inhibitor) and PD146176 (12/15-LOX inhibitor), effectively prevent ferroptosis in various models [34,42]. However, zileuton (5-LOX inhibitor) and PD146176 (12/15-LOX inhibitor) also possess radical-trapping activity, which poses the question of whether anti-ferroptotic activity of these inhibitors is due to LOX inhibition or RTA function [47]. It has been suggested that LOX might provide lipid peroxides at the early phase of ferroptosis, but it is dispensable for the propagation of autoxidation, which might be the actual driver of ferroptotic cell death [47].

### 2.4. Phosphatidylethanolamine-Binding Protein 1 (PEBP1), a Key Regulator of Lipid Peroxidation

Since LOXs primarily catalyze the oxidation of free PUFAs but not PUFAs on phospholipids at the cellular membrane, the mechanism by which LOXs work on membrane phospholipids is unknown. Previously, it was suggested that 15-LOX can bind to PEBP1, which is also known as Raf kinase inhibitory protein (RKIP1), to regulate Raf-1-mediated mitogen-activated protein kinase (MAPK) signaling pathway [48,49]. Based on this observation, Wenzel et al. further revealed that PEBP1 forms a stable complex with 15-LOX and allows 15-LOX to act on PE-associated PUFAs, which results in the generation of 15-hydroperoxyeicosatetraenoic acid-phosphatidylethanolamine (15-HpETE-PE) to induce ferroptosis (Figure 1) [34]. Locostatin, a specific inhibitor of the Raf-1/PEBP1 interaction, increases oxidized PE levels and subsequent ferroptosis upon RSL3 treatment, possibly promoting formation of the 15-LOX/PEBP1 complex [34]. Various models of ferroptosis-related diseases, including asthma, acute kidney injury, and traumatic injury of the brain, were applied, and the authors further showed that 15-LOX/PEBP1 complexes accumulate in the disease state, resulting in an increase in oxidized PEs, such as 15-HETE-PE and 15-HpETE-PE [34].

The importance of PEBP1 in ferroptosis was further emphasized by the suggestion that ferrostatin-1 also targets the 15-LOX2/PEBP1 complex. Established ferroptosis inhibitors, such as Fer-1, liproxstatin-1, and α-tocopherol, are known to exert anti-ferroptotic activity due to their lipophilic radical scavenging properties [16,50]. In contrast, these inhibitors do not inhibit 15-LOX, implying that 15-LOX might be dispensable for lipid peroxidation [51,52]. The ferroptotic activity of the 15-LOX1 inhibitor PD146176 might be due to its radical scavenging activity, raising the question of whether 15-LOX is indeed involved in ferroptosis [51,52]. Since 15-LOX activity was previously measured using free eicosatetraenoic acid (ETE) in the absence of PEBP1, the ability of 15-LOX to oxidize ETE-PE was recently re-evaluated in the presence of PEBP1 [53]. As expected, PEBP1 increases the production of 15-HpETE-PE, an ETE-PE oxidation product, but it does not affect free ETE oxidation [53]. More importantly, Fer-1 suppressed the production of 15-HpETE-PE but not 15-HpETE, suggesting that Fer-1 inhibits the 15-LOX2/PEBP1 complex rather than free 15-LOX2 [53]. Computational molecular modeling suggests that Fer-1 can directly bind to and interfere with the 15-LOX/PEBP1 complex [53]. This study supports that LOXs combined with PEBP1 play a central role in lipid peroxidation and ferroptosis.

### 2.5. Other Oxygenases in Ferroptosis

Given the critical roles of radicals in ferroptosis, other oxygenases, such as NOXs and cytochrome P450 oxidoreductase (POR), are also suggested as crucial contributors to ferroptosis (Figure 1). NOXs generate superoxide radicals from nicotinamide adenine dinucleotide phosphate (NADPH) and oxygen in cells. The canonical NOX inhibitor diphenylene iodonium (DPI) and the NOX1/4-specific inhibitor GKT137831 strongly attenuate erastin-induced ferroptosis in the lung cancer cell line Calu-1 [16]. Since NOX1 is predominantly expressed over NOX4 in Calu-1 cells, NOX1 was suggested as the critical mediator of ferroptosis in these cells [16]. In addition, NOX1 activity is increased by binding with DPP4 in p53^−/−^ or p53-depleted colorectal cancer cell lines [20]. The interaction between NOX1 and DPP4 seems to be crucial in inducing lipid peroxidation in ferroptosis since treatment with the NOX1 inhibitor 2-acetylphenothiazine (2-AC), NOX1 depletion or DPP4 depletion can diminish erastin-induced NOX activity and lipid peroxidation [20]. NOX2 or NOX4 is often activated in ovarian cancer and renal cell carcinoma through the Hippo pathway effector tafazzin (TAZ), promoting erastin-induced ferroptosis [54,55]. Accordingly, depletion of NOX2 or NOX4 prevents erastin-induced ferroptosis [54,55]. Therefore, it seems that specific NOXs play a key role in ferroptosis in a cell type- and context-dependent manner. On the other hand, there remains a debate regarding whether NOXs are indeed required for ferroptosis because NOX inhibitors only partially suppress ferroptosis in some cells and because NOX inhibitors also possess radical scavenging activities [56]. In addition, little is known about whether NOXs are required for RSL3-induced cell death.

POR has also been identified as a contributor to ferroptosis through CRISPR/Cas9-mediated suppressor screening [57]. POR is a membrane-bound enzyme required for electron transfer from NADPH to cytochrome P450 and binds to its cofactors, such as flavin mononucleotide (FMN) and flavin adenine dinucleotide (FAD) [58]. This complex mediates electron supplementation to cytochrome P450 from NADPH and is required for erastin-, FIN56-, ML210-, or RSL3-induced ferroptosis. In addition, POR seems to accelerate the cycling between ferrous and ferric iron in the heme component of cytochrome P450. These processes might directly promote lipid peroxidation [58]. Although LOXs are regarded as primary inducers of lipid peroxidation, their expression is somewhat limited in several types of cancer cell lines. In contrast, POR is broadly expressed in most cancer cells, suggesting the possible crucial role of POR in lipid peroxidation and ferroptosis [58].

An independent genome-wide CRISPR/Cas9 screen also revealed that POR is a key regulator of ferroptosis [59]. In addition to POR, the ER-resident oxidoreductase, cytochrome B5 Reductase 1 (CYB5R1) is required for complete lipid peroxidation [59]. While a previous report showed that cytochrome P450 is an important electron acceptor for ferroptosis, this study suggests that electron acceptors, including cytochrome P450, heme oxygenase (HO-1), and squalene monooxygenase (SQLE), are dispensable for ferroptosis [59]. Rather, POR and CYB5R1 produce H_2_O_2_ by donating electrons to oxygen, triggering iron-dependent Fenton reaction and lipid peroxidation [59].

Activated M1 macrophages and microglia, but not alternatively activated M2 macrophages, are highly resistant to ferroptosis, as they can increase the expression of inducible nitric oxide synthase (iNOS) and induce NO^•^ accumulation in cells [60]. NO^•^ directly reacts with reactive intermediates and lipid peroxyradicals mediated by 15-LOX, forming nitroxygenated lipid species [61]. In addition, NO^•^ also removes secondary lipid radicals, as evidenced by LC-MS data showing nitroxygenated oxidatively truncated derivatives [60]. Understanding the contribution of each enzyme to lipid peroxidation and ferroptosis in various ferroptosis-related diseases will provide a basis for the development of therapeutic agents for diseases through their inhibitors.

### 2.6. Ether Phospholipids in Ferroptosis

While almost all ferroptosis studies focused on lipid peroxidation on diacyl phospholipids, a recent study revealed that ether-linked phospholipids are also critical components for ferroptosis [61]. Genome-wide CRISPR/Cas9 screening was applied in 786-O clear-cell renal cell carcinoma cells and OVCAR-8 ovarian carcinoma cells; several peroxisomal genes, such as peroxisomal biogenesis factor 10 (PEX10), peroxisomal biogenesis factor 3 (PEX3), alkyldihydroxyacetone phosphate synthase (AGPS) and fatty acyl-CoA reductase (FAR1), were identified to be required for ferroptosis [61]. Among the various functions of peroxisomes, the authors focused on ether phospholipids and found that depletion of peroxisomal genes results in the downregulation of ether phospholipids [61]. To directly address whether ether phospholipids are indeed as potent as diacyl phospholipids in terms of inducing ferroptosis, each lipid was delivered to cells using liposomal nanoparticles. Among the PE species, ether-linked PE (ePE) containing AA or docosahexanoic acid (DHA) (ePE-C18:0/C20:4 or ePE-C18:0/C22:6, respectively) sensitized cells to GPX4 inhibitor-induced ferroptosis at a potency similar to that of diacyl PE containing AA (PE-C18:0/C20:4). In addition, ePC-C18:0/C20:4 and ePC-C18:0/C22:6 promoted ferroptosis more potently than PC-C18:0/C20:4. However, phospholipids containing oleic acid (OA; C18:1) did not promote ferroptosis, suggesting that specific PUFAs such as AA and DHA rather than phospholipid species are responsible for ferroptosis induction [61].

Notably, several physiologically important roles of ether phospholipids in ferroptosis were suggested. While most cancer cells depleted of GPX4 fail to grow in vivo, some cells can survive despite a lack of GPX4 expression [61]. These cells contain significantly lower levels of PUFA-containing ether phospholipids (PUFA-ePLs), rendering cells resistant to ferroptosis [61]. Downregulation of PUFA-ePL levels might be a strategy by which tumors evade ferroptosis. The authors also found that the abundance of PUFA-ePLs increases to facilitate the differentiation of neuronal and cardiac progenitor cells into neuronal cells and mature cardiomyocytes, respectively, in vitro [61].

## 3. Pathways That Regulate Lipid Metabolism and Ferroptosis

**Figure 2:**
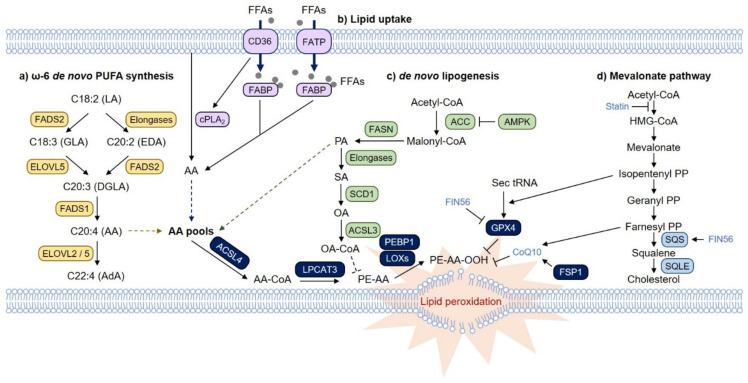
Lipid metabolic pathways that regulate ferroptosis. (**a**) In cells, PUFAs can accumulate through the ω-6 de novo PUFA biosynthesis pathway and fatty acid transport pathways via fatty acid translocase (FAT/CD36), fatty acid transport protein (FATP) and fatty acid binding protein (FABP). Imported linoleic acid (LA) is metabolized by elongation of very long-chain fatty acid protein (ELOVL) and fatty acid desaturases (FADS) to synthesize AA and AdA, which are utilized to produce membrane phospholipids. (**b**) Additionally, AA can be directly imported and transported by FAT/CD36, FATP and FABP proteins. In particular, CD36 activates cytoplasmic phospholipase A_2_ (cPLA_2_), releasing AA from phospholipids. The intracellular pools of AA might be the critical checkpoint in ferroptosis and AA-mediated cellular signaling. (**c**) In contrast, monounsaturated fatty acid (MUFA), which is catalyzed from palmitate, a saturated fatty acid (SFA) by stearyl-CoA desaturase-1 (SCD1), is incorporated into phospholipids in an ACSL3-dependent manner, thereby interfering with the formation of PUFA-phospholipids and protecting cells from ferroptosis. Under glucose deprivation conditions, the depletion of ATPs activate the AMP-activated protein kinase (AMPK) pathway, which suppresses de novo lipogenesis by phosphorylating and inhibiting acetyl-CoA carboxylase (ACC). As a result, the levels of not only palmitate but also dihomo-γ-linolenic acid (DGLA) and AA are reduced upon glucose starvation, preventing ferroptosis. (**d**) The mevalonate pathway also provides several critical regulatory axes. Isopentenyl pyrophosphate (IPP) is required for the isopentenylation of selenocysteine-tRNA, leading to the synthesis of selenoproteins, including GPX4. Inhibition of HMG-CoA reductase (HMGCR) by statins might induce ferroptosis by inactivating GPX4. FIN56, a class III FIN, degrades GPX4 and activates squalene synthase (SQS). Activation of SQS results in the squalene depletion of coenzyme Q10 (CoQ10), contributing to ferroptosis. In contrast, inhibition of SQS leads to squalene depletion and facilitates ferroptosis in lymphoma, as squalene also possesses antiferroptotic activity.

One of the important questions is how cells obtain and maintain the fatty acid pools in cells. Cells are primarily supplied with fatty acids from the blood and lymphatic vessels. While saturated fatty acids (SFAs) and monounsaturated fatty acids (MUFAs) can be generated from acetyl-CoA in cells, PUFAs, which are essential fatty acids, cannot. Instead, long-chain PUFAs such as AA and AdA can be synthesized from dietary essential fatty acids such as linoleic acid (C18:2n-6) and α-linoleic acid (ALA; C18:3n-3) in cells by a series of enzymatic reactions involving fatty acid desaturase (FADS) and elongation of very long-chain fatty acid protein (ELOVL) (Figure 2a) [62]. Alternatively, long-chain PUFAs can also be obtained from outside of mammalian cells. Furthermore, fatty acid uptake and release are tightly regulated by cellular states and external stimuli (Figure 2b). Together, these factors determine the fatty acid pools in cells, which ultimately affect ferroptosis sensitivity.

### 3.1. PUFA Biosynthesis Pathway and Ferroptosis

As mentioned above, AA and AdA are the PUFAs that are most susceptible to lipid peroxidation. AA and AdA are ω-6 fatty acids and can be synthesized from linoleic acid (LA) through the ω-6 de novo PUFA synthesis pathway using ELOVL2, ELOVL5, FADS1 and FADS2 (Figure 2a). These enzymes seem to play an essential role in ferroptosis [63,64]. In particular, intestinal-type gastric cancer (GC) cells express extremely low levels of ELOVL5 and FADS1 due to hypermethylation at the promoter region and are resistant to ferroptosis, while mesenchymal-type GC cells are sensitive to ferroptosis at high levels of ELOVL5 and FADS1 [63]. Isotope-tracing analysis clearly proposed that intestinal-type cells are defective in synthesizing AA and AdA from LA, but these cells become sensitive to ferroptosis upon supplementation with AA [63]. Analysis of The Cancer Cell Line Encyclopedia (CCLE) database suggested that ELOVL5 and FADS1 are expressed in most cancer cells, but those genes are silenced in some types of cancer cells, including gastric and colorectal cancer cells, suggesting that these two enzymes could be used as prediction markers for ferroptosis-mediated cancer therapy [63]. Moreover, depletion or inhibition of FADS2 inhibits RSL3-induced ferroptosis [63,64].

It is curious how just inhibition of PUFA synthesis can block ferroptosis. Transcriptome analysis suggested that there were no significant changes in the levels of fatty acid transporters during this process. Interestingly, we found that FATP2 levels are relatively low in mesenchymal-type GC cells, implying that fatty acid import is somewhat limited in these cells; therefore, these cells are dependent on PUFA biosynthesis [63]. Although AA is regarded as a primary target for lipid peroxidation, ELOVL5-depleted GC cells still contain comparable amounts of AA and PE-AA, possibly activating AA import [63]. Instead, these cells harbor significantly lower levels of AdA and PE-AdA and are resistant to ferroptosis, suggesting that AdA might also be crucial for lipid peroxidation and ferroptosis in GC [63].

### 3.2. MUFAs and Ferroptosis

In contrast to PUFAs, which are essential in ferroptosis, MUFAs, such as OA, can protect cells from ferroptosis (Figure 2c) [17,65]. Lipidomic analysis suggests that OA reduces the amount of ferroptosis-related phospholipids, such as PC- or PE-linked AA or AdA, without altering free AA and AdA levels, suggesting that MUFAs compete with AA and AdA for their incorporation into phospholipids [65]. In this regard, ACSL3, which mediates phospholipid incorporation of MUFAs, is required for MUFA-mediated ferroptosis suppression [65]. Curiously, OA treatment results in the accumulation of free OA but not phospholipid-containing OA, implying that more complex mechanisms underlie the regulation of phospholipid composition and ferroptosis [65].

An interesting observation was that the levels of OA and GSH in lymph fluid were higher than those in blood plasma; the iron levels in lymph fluid were lower than those in blood plasma, and these expression patterns might protect tumor cells from ferroptosis, leading to increased survival rates during metastasis [66]. These results imply that ferroptosis also plays a key suppressive role in tumor metastasis through the blood, but tumor cells metastasizing through the lymph are protected from ferroptosis. Considering that the levels of fatty acids, including MUFAs and PUFAs, are much higher in human serum than in classical culture medium supplemented with fetal bovine serum (FBS), information on how cells maintain free fatty acid pools and phospholipids in cells is much more important for determining whether cells undergo ferroptosis or survive [65,67].

### 3.3. Fatty Acid Transport and Ferroptosis

Fatty acids are imported into cells through various fatty acid transport proteins, such as fatty acid translocase (FAT/CD36), fatty acid transport protein (FATP) and fatty acid binding protein (FABP) (Figure 2b) [68,69]. As membrane phospholipids continuously undergo remodeling, PUFAs are released derived from membrane phospholipids via phospholipase A2 (PLA_2_)-catalyzed hydrolysis. In particular, AA and eicosapentaenoic acid (EPA) are released preferentially by cytoplasmic PLA_2_ (cPLA_2_), whereas DHA is released by Ca^2+^-independent PLA_2_ (iPLA_2_) [70]. Although there are no studies that have directly assessed whether these factors are associated with ferroptosis, numerous studies have provided a potential link between fatty acid transport and ferroptosis.

Recent studies have suggested that chemoresistant tumors with high expression of transforming growth factor-β (TGFβ) and EMT gene signatures are highly vulnerable to ferroptosis [63,71,72]. Furthermore, most malignant tumors often exhibit altered lipid metabolism [73,74]. Moreover, malignant tumors also exhibit increased expression of CD36, which facilitates increased fatty acid uptake from outside of the cell, which promotes the EMT process [75,76]. For example, in prostate cancer, fatty acids imported via CD36 are stored in cellular complexes, such as phospholipids, diacylglycerol (DAG), and triacylglycerol (TAG), rather than being used for fatty acid oxidation [77]. This might facilitate the ferroptosis of malignant tumors overexpressing CD36. In contrast, CD36 has been shown to activate cPLA_2_, thereby releasing AA from phospholipids [78]. Released AA is exported from cells or is converted into prostaglandin E (PGE) [78]. This implies that CD36 is also able to suppress ferroptosis by reducing ferroptosis-related phospholipids, such as PE/PC-linked AA or AdA. Therefore, the role of CD36 in ferroptosis requires further investigation.

Exogenous lipids, including PUFAs and MUFAs, absorbed via CD36 induce metabolic and functional reprogramming of tumor-associated myeloid-derived suppressor cells (MDSCs) [79]. CD36 also directly suppresses the anti-tumor immune function of CD8^+^ tumor-infiltrating lymphocytes (TILs) by promoting lipid peroxidation through the uptake of oxidized low-density lipoproteins (OxLDL) [80]. Ferroptosis might be involved in this process, as GPX4 overexpression can rescue CD8^+^ tumor cell function [80]. Since attempts to induce ferroptosis in cancer cells can also suppress anti-tumor immunity, a strategy to induce ferroptosis specifically in tumors based on the difference in ferroptosis mechanisms between cancer cells and immune cells is needed.

Recently, it was shown that fatty acid transport protein 2 (FATP2) plays pivotal roles in lipid accumulation in polymorphonuclear myeloid-derived suppressor cells (PMN-MDSCs) [81]. In particular, free AA levels are significantly decreased in FATP2 KO cells. AA tracing analysis also revealed that FATP2 KO PMN-MDSCs are defective in the uptake of AA, resulting in lower levels of AA-containing phospholipids and PGE2. Given that PGE2 mediates the tumor suppressive function of MDSCs, FATP2 inhibition might effectively suppress tumor growth through PGE2 [81,82]. On the other hand, restriction of AA uptake by FATP2 deletion is likely to result in ferroptosis resistance. However, while GC cells express low levels of FATP2, cells seem to compensate for AA deficiency by activating the de novo synthesis pathway to sensitize cells to ferroptosis [63].

Recently, the role of iPLA_2_ on ferroptosis was identified by two studies. First, the authors focused on peroxiredoxin 6 (PRDX6) as this enzyme are known to possess phospholipid hydroperoxide activity and iPLA_2_ activity [83]. Depletion of PRDX6 increases RSL3- or erastin-induced ferroptosis with an increase in lipid peroxidation levels suggesting that PRDX6 is a negative regulator of ferroptosis [83]. By employing MJ33, a specific PRDX6 phospholipase A2 (iPLA_2_) inhibitor, the authors propose that iPLA_2_ activity of PRDX6 is responsible for ferroptosis suppression [83]. However, the detailed mechanism how iPLA_2_ remodels membrane phospholipids to suppress ferroptosis is unclear. Another study directly focused on the ability of iPLA_2_β (PLA2G6) to hydrolyze Hp-PE species, which are the main driver of ferroptosis [84]. The authors directly analyze the abundance of 15-HpETE-PE and found that 15-HpETE-PE are upregulated in PLA2G6 KO cells compared to control cells both in normal and RSL3-treated conditions [84]. The authors further show that PLA2G6 KO mice are more susceptible to ferroptosis induced by RSL3 and ischemia/reperfusion (I/R) than wild-type mice during pregnancy, thereby increasing fetal death rates [84].

### 3.4. De Novo Lipogenesis and Ferroptosis

Glucose is the primary energy source of cells and produces ATP through glycolysis. Excess glucose can be converted into fatty acids via the de novo lipogenesis pathway and stored in triglycerides (Figure 2c). In mammals, cells synthesize SFAs such as palmitic acid (PA; C16:0) and MUFAs such as OA (C18:1) from glucose, but cells are defective in generating PUFAs. Glucose deprivation can lead to metabolic stress by depleting ATP, thereby inducing cell death [85]. However, ATP depletion can activate AMP-activated protein kinase (AMPK), relieving energy stress by conserving ATP and promoting cell survival [86]. Recent studies have shown that glucose starvation prevents ferroptosis induced by various stimuli, including cysteine deprivation, GPX4 deletion, erastin, and RSL3 [87]. AMPK-mediated acetyl-CoA carboxylase (ACC) phosphorylation is responsible for ferroptosis suppression by inhibiting the de novo lipogenesis pathway (Figure 2b) [87]. Lipidomic analysis suggests that not only free PA but also various free PUFAs, including dihomo-γ-linolenic acid (DGLA) and AA, are downregulated upon AMPK activation [87]. As an upstream regulator, liver kinase 1 (LKB1, also known as STK11) also suppresses ferroptosis via the LBK1-AMPK-ACC-FAS axis [88]. Since PUFAs cannot be synthesized from PA, AMPK and PA might indirectly affect PUFA pools, thereby inhibiting ferroptosis (Figure 2b).

In contrast, OA, which can be synthesized from PA via stearoyl-CoA desaturase-1 (SCD1), prevents ferroptosis (Figure 2b) [17,66,67], implying that AMPK might suppress ferroptosis via an additional mechanism. AMPK can also support erastin-induced ferroptosis [89]. AMPK is activated by erastin and promotes the phosphorylation of beclin 1 (BECN1) [89]. Phosphorylated BECN1 then inhibits system x_c_^−^ through direct binding to SLC7A11, thereby accelerating ferroptosis [89]. Therefore, understanding how the de novo lipogenesis pathway coordinates with PUFA synthesis pathways to remodel phospholipid metabolism under various conditions will provide insight into lipid peroxidation, ferroptosis, and related diseases.

### 3.5. The Mevalonate Pathway

Since cholesterol can undergo autoxidation and is enriched in cellular membranes and lipoproteins, cholesterol might be associated with ferroptosis [90,91,92]. Cholesterol can be synthesized from acetyl-CoA via the mevalonate pathway (Figure 2d). The mevalonate pathway is also essential for the production of selenoproteins, including GPX4, as isopentenyl pyrophosphate, an intermediate of the mevalonate pathway, is required for the isopentenylation of selenocysteine-tRNA (Figure 2d) [93]. Accordingly, cancer cell line sensitivity data analysis revealed that statins are selective inducers of ferroptosis in mesenchymal-type cancer cells that may act by inactivating GPX4 [72]. Since statins can alter tumor metabolism and suppress cell survival, whether statins indeed specifically induce ferroptosis requires further validation [94]. Another class of ferroptosis inducers, FIN56, activates squalene synthase (SQS) (also known as farnesyl-diphosphate farnesyltransferase; FDFT1) in the mevalonate pathway, which produces squalene from farnesyl pyrophosphate (farnesyl-PP), in addition to inducing GPX4 degradation (Figure 2d) [95]. Since coenzyme Q10 (CoQ10) is synthesized from farnesyl-PP, activation of SQS by FIN56 depletes farnesyl-PP and CoQ10, thereby contributing to ferroptosis (Figure 2d) [95].

Some tumors, such as ALK+ anaplastic large cell lymphoma (ALCL), lose the expression of SQLE, which mediates the generation of cholesterol from squalene, indicating that these tumors are dependent on exogenous cholesterols (Figure 2d) [96]. Interestingly, squalene accumulates in the membrane of these cells, leading to ferroptosis suppression by remodeling membrane phospholipids [96]. Inhibition of SQS, which abolishes squalene accumulation, can sensitize cells to ferroptosis [96]. This result contradicts the findings regarding FIN56-induced ferroptosis in HT-1080 cells; in these cells, inhibition of SQLE or SQS suppresses ferroptosis via the accumulation of farnesyl-PP and CoQ10 [95].

## 4. Conclusions

Ferroptosis is implicated in a variety of human diseases, such as myocardial infarction, atherosclerosis, kidney diseases, liver diseases, and neuronal diseases, as well summarized in recent review articles [22,97,98,99,100]. Numerous ferroptosis inhibitors, including ferrostatin-1, liproxstatin-1, vitamin E analogs, and iron chelators, have been shown to effectively ameliorate disease symptoms in mouse models of each disease [22,97,98,99]. In this review, we discussed the underlying mechanism of lipid peroxidation in ferroptosis and the various lipid metabolic pathways that control lipid peroxidation and ferroptosis. The modulation of lipid metabolism might provide new treatment options for ferroptosis-related diseases. However, as these pathways and related factors have been identified and verified in cancer models, it is largely uncertain whether these pathways are also associated with ferroptosis in various diseases such as cardiovascular disease. For example, ACSL4 is known to be an indispensable factor in ferroptosis, but in our recent study, ACSL4 does not seem to be involved in cardiomyocyte ferroptosis [101]. Therefore, further investigation on how ferroptosis is regulated in various diseases such as cardiovascular diseases is needed.

Ferroptosis induction is an emerging strategy for novel cancer treatments, as ferroptosis can kill cancer cells that are resistant to multiple anticancer drugs. While several FINs, such as RSL3 and erastin, are highly potent in killing cancer cells in vitro, their pharmacokinetic properties, such as their solubility and metabolic stability, are not suitable for in vivo use [24]. However, several FDA-approved drugs, such as sulfasalazine and sorafenib, which also target system x_c_^−^, have been shown to prevent tumor growth in xenograft models [100,102,103]. The biggest obstacle in using FINs for cancer treatment is that other tissues, such as the heart, liver, and kidney, are also vulnerable to ferroptosis, which can lead to undesirable side effects. In addition, ferroptosis can affect tumor-suppressing immune cells, thereby limiting anti-cancer immunity. Therefore, to effectively target this process, a cancer-specific ferroptosis induction strategy is necessary to efficiently treat tumor patients.
